# Chitosan/Pluronic F127 Thermosensitive Hydrogel as an Injectable Dexamethasone Delivery Carrier

**DOI:** 10.3390/gels8010044

**Published:** 2022-01-07

**Authors:** Jomarien García-Couce, Miriela Tomás, Gastón Fuentes, Ivo Que, Amisel Almirall, Luis J. Cruz

**Affiliations:** 1 Biomaterials Center, University of Havana, Avenida Universidad entre G y Ronda, Vedado, Plaza, La Habana 10400, Cuba; J.Garcia_Couce@lumc.nl (J.G.-C.); amisel@biomat.uh.cu (A.A.); 2 Translational Nanobiomaterials and Imaging Group, Department of Radiology, Leiden University Medical Center, 2333 ZA Leiden, The Netherlands; I.Que@lumc.nl; 3 Unidad de I + D, Empresa Laboratorios AICA, La Habana 11300, Cuba; miriela9005@gmail.com

**Keywords:** chitosan, pluronic, thermosensitive hydrogels, dexamethasone, intra-articular injection, osteoarthritis, cartilage

## Abstract

Intra-articular administration of anti-inflammatory drugs is a strategy that allows localized action on damaged articular cartilage and reduces the side effects associated with systemic drug administration. The objective of this work is to prepare injectable thermosensitive hydrogels for the long-term application of dexamethasone. The hydrogels were prepared by mixing chitosan (CS) and Pluronic-F127 (PF) physically. In addition, tripolyphosphate (TPP) was used as a crosslinking agent. Chitosan added to the mix increased the gel time compared to the pluronic gel alone. The incorporation of TPP into the material modified the morphology of the hydrogels formed. Subsequently, MTS and Live/Dead^®^ experiments were performed to investigate the toxicity of hydrogels against human chondrocytes. The in vitro releases of dexamethasone (DMT) from CS-PF and CS-PF-TPP gels had an initial burst and took more time than that from the PF hydrogel. In vivo studies showed that hydrogels retained the fluorescent compound longer in the joint than when administered in PBS alone. These results suggest that the CS-PF and CS-PF-TPP hydrogels loaded with DMT could be a promising drug delivery platform for the treatment of osteoarthritis.

## 1. Introduction

Osteoarthritis (OA) is a chronic degenerative disease characterized by the gradual loss of cartilage and synovial inflammation. It primarily affects weight-bearing joints [1]. Its main symptomatology is severe pain that causes loss of joint function, leading to the disability of individuals who suffer from it [2,3]. It is a highly prevalent disease worldwide that affects more than 250 million people, which is a figure that continues to increase due to the aging population and the increasing life expectancy [4]. Despite the studies carried out to date, it has not been possible to find a treatment that that can cure OA. Until now, treatments have been aimed at reducing the main symptomatology, with the use of anti-inflammatory drugs by oral and/or topical vial [5]. However, the frequent administration of these drugs by oral vial causes serious gastrointestinal side effects, and the effectiveness of topical applications is very low. Another pharmacological treatment that has been gaining space is the administration of corticosteroids directly to the damaged joint. This reduces systemic side effects [2,4]. Within this pharmacological group, there are five corticosteroids approved by the United States Food and Drug Administration (FDA) for intra-articular administration (IA) [2,6]. One of them is dexamethasone (DMT), which is a glucocorticoid with an anti-inflammatory potency 20 to 30 times greater than natural hydrocortisone and whose pleiotropic protective properties make it an attractive candidate for IA application [2,7]. In in vitro and in vivo studies developed by several authors, they demonstrated that the application of DMT reduces the loss of proteoglycans and collagen from the extracellular matrix, maintains matrix synthesis, and preserves chondrocyte viability [5,6,8]. Furthermore, it is a key reagent for inducing mesenchymal stem cell chondrogenesis in vitro [5,9].

Unfortunately, these molecules are quickly eliminated from the application site due to lymphatic drainage requiring repeated administration that is detrimental to the well-being of the patient [1]. Research to achieve formulations that allow a longer residence time of the active molecules in the joint has been constant and intense. However, so far, there is only one extended-release formulation for intra-articular application approved in 2017 by the FDA and available on the market under the name Zilretta. This formulation is an injectable suspension of triamcinolone acetonide encapsulated in polylactic-co-glycolic acid (PLGA) microparticles that allows the action to be extended for 12 weeks. Although we might think that it is the ideal formulation, it has disadvantages: one of them is its high cost, which exceeds 600 UDS [10], in addition to the fact that the efficacy and safety of repeated administration has not been demonstrated [11]. Therefore, the search for intra-articular formulations continues to be a field of great interest in the science of biomaterials. Injectable hydrogels have acquired great relevance in recent times due to their in situ gelling properties [12]. These hydrogels present a sol–gel transition due to physiological changes, such as temperature, and form a deposit that releases the drug in a controlled manner and prolongs its permanence within the joint [1,13].

In situ gelation by temperature action can be achieved using thermosensitive polymers that form a gel by sensing body temperature. The family of pluronics or polaxomers has the ability to form gels at temperatures close to body temperature. This is why they have become important polymers in the field of injectable hydrogels [14,15]. Due to their characteristic triblock structure poly(ethylene oxide)–poly(propylene oxide)–poly(ethylene oxide) (PEO—PPO—PEO) that consists of hydrophilic (PEO) and hydrophobic (PPO) segments, these polymers form micellar structures formed by the PPO core and the PEO crown under the action of temperature [16,17]. Subsequently, these micelles self-assemble into ordered cubic or hexagonal phases, producing thermoreversible hydrogels [16,18]. Pluronic F-127 (PF) has a molecular weight of 12.6 KDa and has been the most widely used of this family of copolymers for applications in controlled release systems due to its low immunogenicity and toxicity [14,19]. However, PF hydrogels maintain the drug release for a short period due to their rapid dissociation in aqueous medium [20]. One of the ways to modify this condition is to physically mix them with other polymers to alter the release of the drug [21]. Various additives such as hyaluronic acid [22,23], chitosan [24,25], and alginates [26,27] among others have been widely used by researchers to modulate the poloxamer release profile based on in situ gelation systems.

Chitosan (CS) is a natural cationic polysaccharide that has gained great prominence as a candidate for joint cartilage repair because it is similar to glycosaminoglycans present in the extracellular cartilage matrix (ECM) [28,29]. CS is obtained mainly by the partial deacetylation of chitin, which is the second most abundant natural polysaccharide; it is mainly obtained as a subproduct of shellfish, such as crabs and shrimp [29,30]. It has excellent biocompatibility, biodegradability, and non-toxicity properties, as well as excellent bioadhesive properties due to the presence of positive charges in its structure [31,32].

Based on the above, the main objective of this work is to develop a thermosensitive hydrogel from the physical mixture of CS and PF for the controlled release of dexamethasone. Although this physical combination of polymers has been used previously by other authors for intranasal [14,25], vaginal [33], intravesical [34], ocular [35] delivery, etc., there are no reports of its use for intra-articular application. The hydrogels prepared combine the thermogelling properties of PF with the properties of biocompatibility, bioadhesiveness, and structural similarity with glycosaminoglycans that chitosan has.

## 2. Results and Discussion

In this work, different hydrogel samples were prepared varying the concentration of chitosan and pluronic in the mixture, as summarized in Table 1. The rest of the parameters for the preparation were kept constant. The amount of PF corresponding to each sample was dissolved in the chitosan solution, leaving a totally transparent solution. This shows that the components were mixed homogeneously. However, the CS2-PF25 sample could not be completed because PF was not completely dissolved.

### 2.1. Hydrogel Characterization

#### 2.1.1. Gelation Time

**Figure 1:**
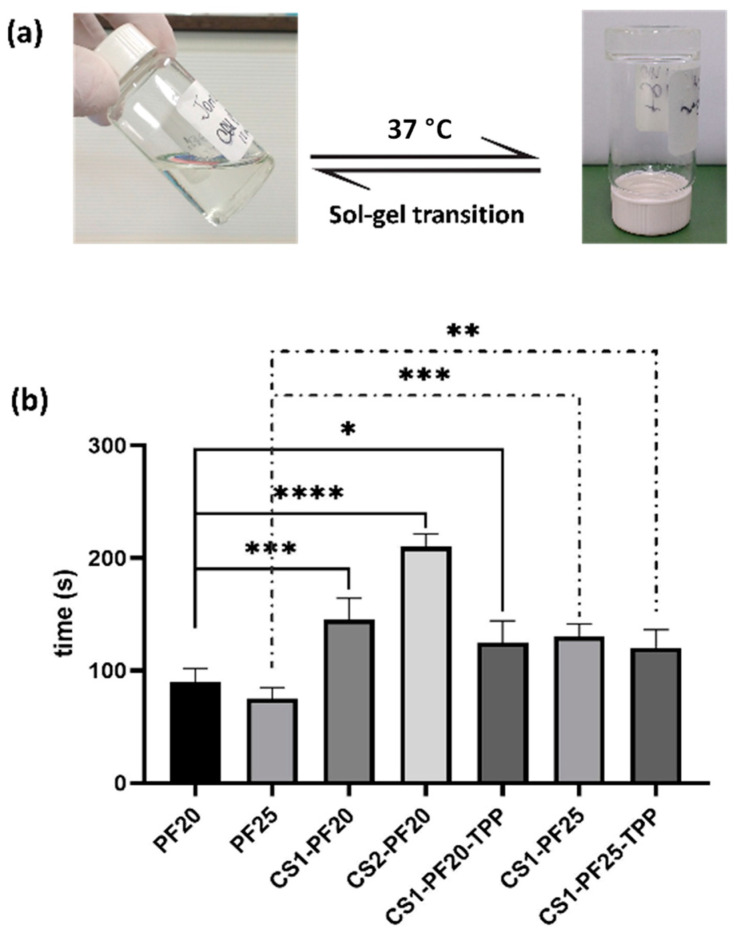
The sol–gel transition characteristics of a thermosensitive gel: (**a**) Phase changes with a temperature increase. (**b**) Gelation time of CS-PF hydrogels containing different CS and PF concentrations, with and without TPP. Values represent mean ± SD (*n* = 5). All experiments were performed five times. (Ordinary one-way ANOVA, * *p* < 0.05, ** *p* < 0.01, *** *p* < 0.001 and **** *p* < 0.0001).

The first characteristic to be evaluated in these materials is the gelation time: that is, the time necessary for the material in the sol phase to form the gel at the physiological temperature of 37 °C (Figure 1a). This time must be sufficient to allow all products to be administered at the specified site in liquid form (sol phase) and the transition to gel to occur for the material not to leave the application site.

The vial tilting method was used to visualize gelation with temperature change by measuring the flowability of the prepared hydrogels. As can be seen in Figure 1b, the increase in pluronic and chitosan concentrations modified the gelation time. For the samples evaluated as control (PF20 and PF25), which are only PF at 20 and 25% (*w*/*v*) respectively, the increase in concentration reduces the time of gel formation. This is to be expected, as there is a greater number of triblock chains that favor the formation and packaging of micelles [36,37]. However, the addition of chitosan to the mixture produces the opposite effect: it increases the gelation time, because the chitosan chains are interspersed, which reduce the hydrophobic interactions of PF molecules. This allows the formation and packaging of micelles to form the gel [38,39,40]. On the other hand, the addition of TPP did not introduce significant changes, but it slightly reduced the transition time in both formulations. This is due to the crosslinking process that occurred between CS and TPP, which facilitated the gelling process [41]. Now, although all the samples had a gelation time within an appropriate range, CS1-PF25 and CS1-PF25-TPP samples were selected for subsequent studies, since the gel formed was more stable and provided better conditions for the matrix used as a delivery system.

#### 2.1.2. Morphological and Chemical Characterization of Hydrogels

**Figure 2:**
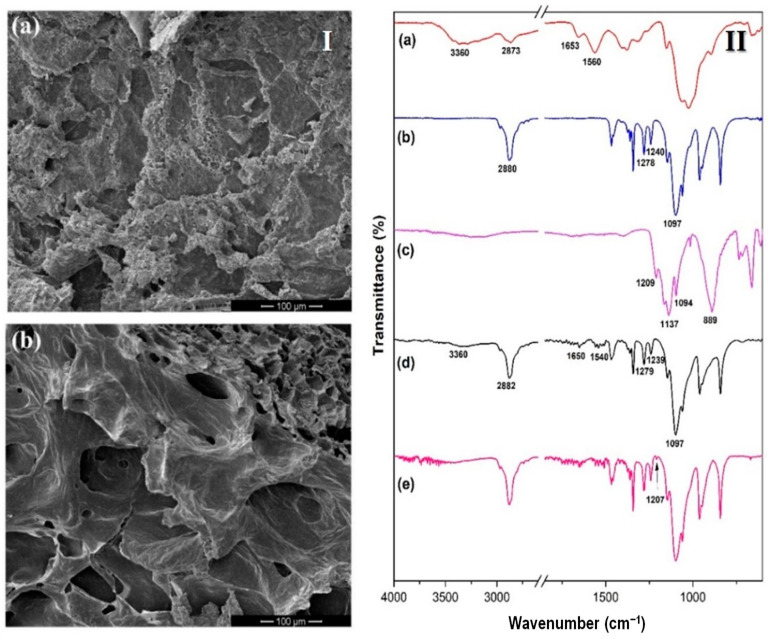
(**I**) SEM micrographs of freeze-dried hydrogels, CS1-PF25 (**a**) and CS-PF-TPP (**b**). (**II**) FTIR spectra of CS (**a**), PF 127 (**b**), TPP (**c**), CS-PF hydrogel, and CS-PF-TPP hydrogel ((**d**,**e**), respectively).

The morphology of the selected hydrogels formulations, previously lyophilized, is shown in Figure 2I. In the micrograph (Ia), it is observed that the morphology of CS1-PF25 hydrogel has a heterogeneous and irregular structure, with the presence of channels that cannot be exactly defined as pores; however, they are areas favoring the exchange of fluids between the matrix and the surrounding environment. This morphology is due to the high concentration of PF in the sample, which implies a high number of hydrophobic moieties in the hydrogel [42]. On the other hand, as expected, the morphology of the CS1-PF25-TPP sample (micrograph Ib) is different compared to the morphology observed for the CS1-PF25 sample due to the addition of the crosslinking agent, TPP. The presence of the crosslinker in the matrix causes an entanglement of the CS chains, approaching each other. This generates the presence of pores in the structure, as clearly observed. The increase in porosity observed in the second sample is also favorable for controlled release systems [41].

To obtain information about the composition of the prepared materials, the spectra of the initial components and the lyophilized hydrogels were recorded. Figure 2II shows the spectra of chitosan, pluronic, and TPP as well as of CS-PF and CS-PF-TPP hydrogels. Figure 2II(a) shows the spectrum of chitosan with its characteristic signals: a broad absorption pick between 3500 and 3200 cm^−1^ attributed to the stretching vibration of O–H and N–H as well as another more discrete signal around 2900 cm^−1^ associated with the stretching vibration of CH. On the other hand, signals are observed at 1653 cm^−1^ attributed to the stretching vibration of C═O in the amide I group and at 1560 cm^−1^ attributed to the N–H bending and the C–N stretching vibrations in the amide II group [43,44,45]. The spectrum of Figure 2II(b) corresponding to pluronic shows the signals associated with the functional groups present in this polymer. Some strong signals can be observed corresponding to the stretching vibrations of different bonds: 2880 cm^−1^ (C–H stretch), 1278 and 1240 cm^−1^ (C–O–C stretches), and 1097 cm^−1^ (C–O stretch) [46,47,48]. On the other hand, in the TPP spectrum (Figure 2II(c)), it is possible to observe absorption bands at 1208 cm^−1^ belonging to the stretching vibrations of P═O, at 1137 cm^−1^ symmetric and antisymmetric stretching vibrations of the PO_2_ group, at 1094 cm^−1^ symmetric and antisymmetric stretching vibrations of the PO_3_ group, and at 889 cm^−1^ antisymmetric stretching vibrations of the P–O–P bond [49,50]. When d and e spectra belonging to the hydrogels prepared are compared with the a–c spectra of the initial components, it is clearly noted that although the characteristic signals of both initial polymers are observed, their largest amount corresponds to PF, which has the highest proportion in the final material. In the spectrum of the CS-PF-TPP hydrogel, a small additional band is observed at 1207 cm^−1^, which is a signal that is associated with the antisymmetric stretching vibrations of the PO_2_ groups in the TPP ions. It indicates the formation of ionic crosslinks between chitosan protonated amino groups and tripolyphosphate anionic groups. This behavior has been observed by other authors in previous works to obtain different materials of chitosan crosslinked with TPP [51,52,53,54,55].

### 2.2. In Vitro Cell Experiments

**Figure 3:**
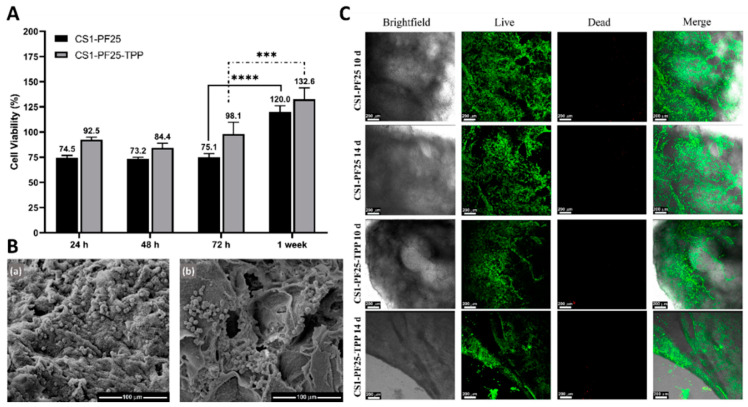
Cell viability assay and cell morphology of cells grown on hydrogels. (**A**) Cell viability of C28/I2 cells grown on CS1-PF25 and CS1-PF25-TPP hydrogels (two-way ANOVA, Tukey’s multiple comparisons test *** *p* < 0.001 and **** *p* < 0.0001). (**B**) SEM images of chondrocytes cultured for 3 days on CS1-PF25 (**a**) and CS1-PF25-TPP (**b**) hydrogels, the scale bar corresponds to 100 μm. (**C**) Live/Dead^®^ staining images of chondrocytes seeded on CS1-PF25 and CS1-PF25-TPP hydrogels after the 10th and 14th day, the scale bar corresponds to 200 μm.

Any hydrogel that can be used as a biomaterial must have excellent biocompatibility. It should not induce cytotoxic effects and should favor cellular processes such as adhesion, growth, and proliferation [56]. Figure 3 shows the results obtained from the MTS and LIVE/DEAD^®^ assays as well as the micrographs of the cells seeded on the materials.

In Figure 3A, it can be seen that the presence of the crosslinking agent in the concentration used did not have adverse effects on cells. On the contrary, the cell viability remained similar, and it was even slightly higher compared to the hydrogel without TPP. During the first three days, cell viability was higher than 70% in both hydrogels; so, according to the ISO 10993-5 standard, we can say that they were cytocompatible against C28/I2 chondrocytes. According to the statistical analysis, there were no significant differences between the values of the number of alive cells during the first three days, but there were when comparing the percentages of cell viability between the third and seventh days. In addition, it is observed that after one week of incubation, the cell viability values were higher than 100%, which shows that the materials, apart from being cytocompatible, also allow cell proliferation.

On the other hand, the morphology of the C28/I2 cells was analyzed after being seeded on the surface of the hydrogels for 72 h. Cell shape plays an important role in the phenotypic expression of chondrocytes. The rounded morphology indicates characteristics of the chondrogenic phenotype [57,58]. Figure 3B shows the SEM micrographs of the cells seeded in the materials, (a) CS1-PF25 and (b) CS1-PF25-TPP. As can be seen in both cases, the cells adhered to the surface of the materials, maintaining their spheroid morphology, and cells with abnormal shapes were not observed. This indicates that the materials allow the maintenance of the normal morphology of chondrocytes, maintaining its chondrogenic characteristics.

The Live/Dead^®^ assay (Figure 3C) visualizes the alive and dead cells in green and red colors respectively, as well as how they are distributed on the material after 10 and 14 days of incubation. For both hydrogels, it can be observed in Figure 3C that the presence of dead cells is very low in both incubation times. The alive cells were homogeneously distributed on the evaluated materials, demonstrating that there was a good material–cell interaction. If otherwise the number of dead cells would be higher, this is because if the seeded cells cannot properly interact with the biomaterial, they will not attach to it and will finally die [56]. Overall, these three results analyzed together indicate that both hydrogel samples were able to favor the adhesion, proliferation, and growth over time of C28/I2 chondrocytes.

### 2.3. In Vitro DMT Release Study

**Figure 4:**
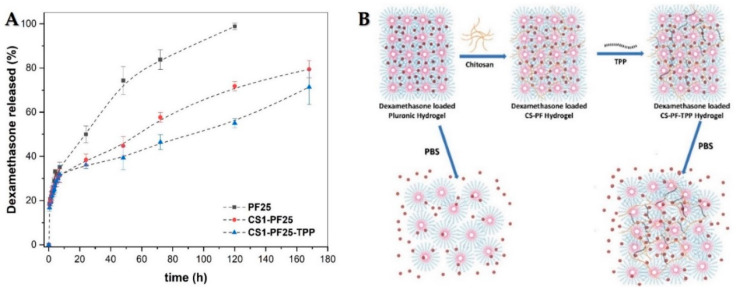
(**A**) Release behavior of dexamethasone from loaded PF25, CS1-PF25, and CS1-PF25-TPP hydrogels. (**B**) Schematic representation of dexamethasone release behavior from CS1-PF25 and CS1-PF25-TPP hydrogels.

In order to evaluate the influence of the samples’ composition on DMT released from PF25, CS1-PF25, and CS1-PF25-TPP hydrogels, in vitro release assays by UV–vis absorbance were carried out in phosphate buffer solution (pH 7.4 and 37 ± 0.5 °C). The profiles of the amounts of dexamethasone released during 7 days are shown in Figure 4A.

As it frequently happens in delivery systems, an initial burst was observed during the first 8 h of the study in which approximately 35% of the occluded dexamethasone was released both in the pluronic and CS-PF mixed hydrogels. These results are common, as the drug closer to the surface can escape more easily in contact with the surrounding fluid due to the rapid interaction. Furthermore, in this particular case, the released drug is highly soluble and has a low molecular weight, which further favors the release. From this time onwards, a difference between the amount of dexamethasone released from the PF hydrogel and from the prepared CS-PF hydrogels is observed. In the PF hydrogel, the release occurs mainly due to the disentangled rate of the micelles that leads to a dissolution of the gel. The drug is left free in the PBS solution [59]. The decrease in the release of dexamethasone from CS1-PF25 and CS1-PF25-TPP hydrogels was due to the reduction of the diffusion rate of the micellar aggregates of PF trapped within the compartments of the chitosan networks [20]. As was also expected, in the sample crosslinked with TPP, the release was lower due to the fact that the interlaced chitosan chains managed to retain even the micellar aggregates of PF. The swelling processes are reduced, and therefore, the release of dexamethasone is even more controlled. In the scheme shown in Figure 4B, we represent graphically how the release process can be modified according to the composition of the hydrogels under study.

The empirical mathematical models described in the literature relate that the amount of drug released is a function of time. Having experimental data, it is possible to look for which model or models it fits and, based on this, explain the possible mechanisms that condition the exit of the bioactive agent from the matrix under study.

In our work, four different kinetic models were used to identify the release mechanism of DMT from the hydrogels prepared. Table 2 shows the results of the adjustments made. Considering the determination coefficients obtained, it was evaluated whether the experimental data conformed to the proposed models and, based on this, an analysis of the rest of the parameters in each equation was carried out. The adjustments made with the model proposed by Higuchi, which describes a release mechanism conditioned by a Fickian-type diffusion [60,61], were not adequate. This shows that the release of the drug is not mainly conditioned by a Fickian-type diffusion.

Next, an analysis was carried out applying the models proposed by Korsmeyer–Peppas and Lindner–Lippold, the first of which establishes an exponential relationship between release and time. It was developed specifically for the release of molecules from polymeric matrices such as hydrogels [62,63]. The second one incorporates a new term into that same equation considering the burst effect that is frequently observed in the release processes from this type of matrixes. It is directly linked to the release of the drug closest to the surface and releases more easily from the matrix. The results in Table 2 show that for all the matrices evaluated, the correlation coefficient for both models was higher than 0.97, with which we can say that the experimental results fit both models satisfactorily. As for the exponent *n*, called the release exponent, it gives us information based on the value it reaches. In the results obtained when applying the Korsmeyer–Peppas model, it is observed that the *n* values are less than 0.5. It implies that a quasi-Fickian type process occurs in which other factors are influencing in addition to diffusion, which are associated with the characteristics and nature of hydrogels. One of the processes that is occurring and can be seen in the graph of the release profiles in Figure 4A is the burst effect, which is taken into account in the Lindner–Lippold model [64]. When we analyze the values of *n* and *b* that result from the fit of the experimental data to the model, we note that the values of *n* increase approaching 0.5. In the PF25 hydrogel used as comparison, the value of *n* was 0.49 and in turn shows the greater value of *b*. These results allow us to conclude that the burst effect is the main factor that is influencing the DMT release process from the PF25 matrix in addition to the diffusion process. When we then analyze the results of CS1-PF25 and CS1-PF25-TPP hydrogels, we see that the values of *K*, *n*, and *b* do not have a notable difference. Thus, it can be deduced that the burst effect is influencing the release of DMT in a similar way from both hydrogels, although it is not the only factor. In the release profiles, there are differences between the DMT released from CS1-PF25 and CS1-PF25-TPP after 24 h of study. For this reason, the fit to the Peppas–Sahlin model was also carried out, considering the process of Fickian diffusion and the relaxation of the polymeric chains [65]. The correlation coefficients show a fit of ≈0.99 in all materials. Now, when we analyze the diffusion (*K_1_*) and relaxation (*K_2_*) constants in the case of CS1-PF25 and CS1-PF25-TPP hydrogels, the values of *K_1_* are very similar, showing that the effect of diffusion on the DMT release affects both matrices in a similar way. The *K_2_* values are small, indicating that the effect of chain relaxation is present in the DMT release process, although to a lesser extent than the diffusion process. However, we can note that the *K_2_* values obtained for the CS1-PF25 and CS1-PF25-TPP hydrogels differ by an order of magnitude, being lower for the CS1-PF25-TPP hydrogel. This reinforces our previous analysis of the effect of the crosslinking of the matrix on the release process.

### 2.4. In Vivo Images of the Injected Hydrogels

Knowing the time of permanence of materials in vivo in the joint after being injected is highly important for its function as a platform for controlled drug release. The longer the length of stay, the better the treatment results that could be achieved.

**Figure 5:**
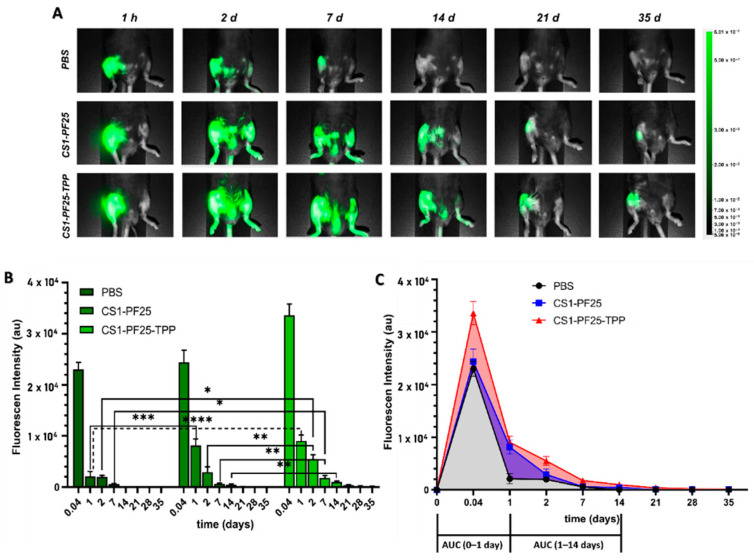
In vivo retention of CS-PF hydrogels: (**A**) NIR fluorescence imaging of joint of mice taken at different times after intraarticular injection with PBS + NIR 780, CS1-PF25 + NIR 780, and CS1-PF25-TPP + NIR 780. (**B**) In vivo retention over time of the various CS-PF hydrogels formulations indicated by relative fluorescence intensity. (**C**) Graphic of area under the curve (AUC) of the three studied groups. Data are presented as mean ± SD. Statistical significance was determined by two-way ANOVA with multiple comparisons; *p < 0.05, ** *p* < 0.01, *** *p* < 0.001, and **** *p* < 0.0001.

After two weeks of induction of osteoarthritis, the materials were applied by intra-articular injection in the mice, and they were followed for 35 days, taking the measurements at the preset times. A representation of the fluorescence images obtained from each group is shown in Figure 5A. For all images, high levels of NIR fluorescence were observed at the articular knee joint shortly after injection, and diffusion was apparent after maximum levels in NIR fluorescence were reached. After this point, the intensity and area of fluorescence gradually decreased. It can be noted that in the control mice that were injected with NIR in only PBS on the second day, a decrease in fluorescence intensity is observed; after 7 days, low NIR fluorescence was observed, and no fluorescence was observed after 14 days.

However, in the samples treated with the hydrogels, a prolonged fluorescence was observed, which gradually decreased over time and could even be seen slightly after 35 days. In the intensity graph (Figure 5B), the reduction in fluorescence that occurs over time is observed in more detail. With the CS1-PF25-TPP hydrogel, the intensity was reduced more slowly over time even compared to non-crosslinked hydrogel.

Additionally, a y–x type graph (fluorescence intensity vs. time) was made to make a more quantitative analysis from the determination of the area under the curve (AUC) of the graphs obtained. In Figure 5C, the area corresponding to each curve can be seen in different colors. To complement Figure 5C, Table 3 shows the values of the total area under the curve for each group of mice and also the area in different sections of the graph (represented also in Figure 5C).

As can be seen, the area of the graph corresponding to the mice injected with the CS1-PF25-TPP hydrogel is the largest. If we take this group as a reference and assume it as 100%, we determine that the group treated with the CS1-PF25 hydrogel showed an average intensity value of 71.2% with respect to the reference group, while for the PBS group, it was 53.6%. Now, if we applied this same analysis carried out to the global graph to the two fractions selected and shown in Table 3, we could see how that percent value with respect to the group that we selected as a reference decreases. In the case of the two measurements carried out within the first day, it can be seen that the area for the group treated with the CS1-PF25 hydrogel represents 74.7% and that for the PBS group represents 63.1%.

However, the difference becomes even greater in the interval between day 1 and day 14 where the percent representing the area under the curve of the graphs corresponding to the group treated with the CS1-PF25 hydrogel and with the NIR solution in PBS was 63.6% and 29.1%, respectively. All this analysis corroborates that the formulations of the hydrogels are retained for a longer time within the implantation site and, furthermore, the TPP added as a crosslinking agent improves the benefits of these materials. Taken together, these results reinforce the hypothesis that the hydrogels prepared are suitable materials to control the release of molecules trapped in their interior and also favor permanence in the application site.

## 3. Conclusions

In this work, thermosensitive hydrogels, consisting of CS and PF and also the crosslinking agent, TPP, were prepared and characterized as a platform for the intra-articular application of DMT. The in vitro results show that the incorporation of chitosan and TPP favors a controlled release of DMT that is spread over a longer time compared to the DMT released from the PF hydrogel alone. Additionally, the fit of the release profiles to different mathematical models allowed us to identify that the processes involved in the release are the initial burst effect, quasi-Fickian diffusion, and to a lesser extent the relaxation of the chains. Furthermore, the biocompatibility of the hydrogels was confirmed by cell viability and the proliferation of human chondrocyte C28/I2 using MTS and Live/Dead^®^ assays. The in vivo study showed that the prepared hydrogels remained for a long time at the injection site and also delayed the release of the fluorescent compound included for its follow-up by imaging. This shows that it can control the release in vivo of occluded molecules. Therefore, we consider these dexamethasone-loaded injectable thermosensitive hydrogels to be promising materials for intra-articular application, and they have potential for the localized treatment of damaged articular cartilage.

## 4. Materials and Methods

### 4.1. Materials

Commercial Pluronic F127 (PF) (M.W. = 12,600), chitosan (CS) from shrimp shells (low viscosity), and sodium tripolyphosphate (TPP) 85% were provided by Sigma-Aldrich (San Luis, MO, USA). Dexamethasone sodium phosphate was obtained from Crystal Pharma (Valladolid, Spain). Phosphate-buffered saline (PBS) was produced by Fresenius Kabi GmbH (Graz, Austria). Dulbecco’s phosphate buffered saline (DPBS), Dulbecco’s modified eagles’ medium (DMEM, high glucose, with Glutamax^TM^), fetal bovine serum (FBS), penicillin, and streptomycin were purchased from Life Technologies (Breda, The Netherlands). 3-(4,5-Dimethylthiazol-2-yl)-5-(3-carboxymethoxyphenyl)-2-(4-sulfophe-nyl)-2H-tetrazolium (MTS, Promega, Madison, WI, USA) and a calcein-AM/ethidium homodimer-1 LIVE/DEAD^®^ assay kit (Invitrogen) were obtained from Carlsbad, CA, USA.

### 4.2. Preparation of CS-Pluronic F-127 (CS-PF) Hydrogel

Thermosensitive chitosan–pluronic hydrogels were prepared from the physical mix of a chitosan (CS) solution with a prefixed mass of Pluronic F127 (PF). The chitosan solution was prepared in acetic acid (1% *v*/*v*) aqueous solution under moderate mechanical stirring for 24 h. A pre-gel solution of CS-PF was prepared according to the “cold method” described by Schmolka [66]. Briefly, PF was added to the cold CS solution slowly over a period of about 5–10 min with gentle stirring by magnetic stirrer (IKA^®^-RH basic 2, Staufen, Germany). Then, the polymer solution was left overnight at 4 °C to ensure the complete dissolution of PF; the pH of the final solution was 6.23. CS-PF hydrogels crosslinked with sodium tripolyphosphate (CS-PF-TPP) were also prepared. First, 550 μL of 0.01 M TPP solution was added to the physical mix of CS-PF under continuous stirring at 4 °C. Table 1 shows the composition and nomenclature of the hydrogels.

### 4.3. Hydrogel Characterizations

#### 4.3.1. Hydrogel Gelation Time

The sol-gel phase transition behavior or the time to gel (indicated as gelation time) of the CS-PF aqueous solutions was investigated using the vial tilt method. Gel time was determined considering the state of the gel when the solution did not flow for 1 min after inverting a vial [67,68,69]. The CS-PF solution prepared (5 mL) was taken up in a glass vial and heated in a water bath at 37 °C, allowing it to gel. After each minute, the glass vial was removed and inverted for 1 min to check the gelling of the sample. The CS-PF hydrogel crosslinked with TPP was prepared in the same manner as described before; the TPP solution was added at the same time in the vial before incubating at 37 °C.

#### 4.3.2. Morphology and Chemical Composition of CS-PF Hydrogels

In order to characterize the morphology and structure of the freeze-dried CS-PF hydrogels, scanning electron microscopy (SEM) was employed. For SEM analysis, CS-PF hydrogels specimens were cross-sectioned. They were mounted on metallic stubs, sputter-coated with Pt/Pd, and their surface morphology was observed in a NanoSEM 200 microscope (FEI, Tokyo, Japan) at the accelerating voltage of 10 kV. The chemical groups of the hydrogels were examined by Fourier transform infrared spectroscopy (FTIR, Kyoto, Japan). The freeze-dried hydrogel samples were analyzed in attenuated total reflectance (ATR) mode using a Shimadzu IRSpirit-T FTIR spectrophotometer (Kyoto, Japan). FTIR analysis was performed ranging from 4000 to 400 cm^−1^ using 32 scans and a resolution of 4 cm^−1^ [70].

### 4.4. In Vitro Experiments

#### 4.4.1. Cytotoxicity Evaluation (MTS Assay)

Human chondrocytes C28/I2 were used as model cells to carry out the in vitro cellular studies. Cells were cultured in 75 cm flasks in Dulbecco’s Modified Eagles’ Medium (DMEM) supplemented with fetal bovine serum (FBS) and 1% antibiotics (penicillin–streptomycin) at 37 °C and an atmosphere of 95% air and 5% CO_2_.

To determinate the cytocompatibility of CS-PF and CS-PF-TPP hydrogels against C28/I2 cells, a 3-(4,5-dimethylthiazol-2-yl)-5-(3-carboxymethoxyphenyl)-2-(4-sulfophe-nyl)-2H-tetrazolium (MTS) assay was performed. A piece of dried hydrogel sample was placed in a 96-well plate, and cell suspensions (1 × 10^4^ cells/well) in Dulbecco’s modified Eagles medium (DMEM) were seeded on the hydrogel. To establish the cytotoxicity controls, cells treated with 50% DMSO and non-treated were used as positive and negative controls respectively. The MTS assay was performed at 1, 2, 3, and 7 days. At each time point, 20 µL of MTS was added and incubated in darkness for 3 h. After that, 100 µL of the supernatant was extracted to a new 96-well plate. The absorbance of each well was measured by a micro-plate reader (VersaMax equipped with Softmax Pro, Molecular Devices, San José, CA, USA) at 490 nm. Cell viability is expressed as the percentage of cells alive in relation to negative control (cells incubated only with DMEM culture medium were used as a negative control (100%)).

#### 4.4.2. Cell Morphology Study

The cell adhesion test was performed by seeding the C28/I2 cells at a concentration of 3 × 10^4^ cells/well on the freeze-dried hydrogel samples. The samples were incubated 37 ± 2 °C with 5% CO_2_ in a humidified atmosphere for 72 h. After this time, the samples were fixed with 2.5% glutaraldehyde for 1 h and subsequently rinsed with DPBS. Then, the samples were dehydrated with an ethanol gradient (60%, 70%, 80%, 90%, and 100%) for 10 min at each concentration, and they were dried in hexamethyldisilazane (HMDS) [71]. Finally, the samples were coated with Pt/Pd and studied in a scanning electron microscope, as previously described above (3.3.2).

#### 4.4.3. Cell Viability (LIVE/DEAD^®^ Assay)

The viability of C28/I2 cells against hydrogels samples was also tested by a LIVE/DEAD^®^ assay performed 10- and 14-days post culture. The C28/I2 cells were seeded on top of the hydrogels (a total of 3 × 10^4^ cells/well) in a 48-well plate and incubated for 10 and 14 days. Then, the supernatant was removed. Hydrogels were washed three times with PBS, and an aliquot of the assay solution containing 4 µM EthD-1 (ethidium homodimer-1) and 2 M calcein AM was added. After 30 min incubation at room temperature, the samples were observed using a confocal microscope (Leica TCS SP5, Leica Mycrosystems BV, Amsterdam, The Netherlands) with excitation filters of 450–490 nm (green, Calcein AM) and 510–560 nm (red, EthE-1). Living cells were observed in green and dead cells were observed in red.

#### 4.4.4. Drug Release Study

A dexamethasone release study was carried out in 5 mL tubes from the charged hydrogels, and a 25% pluronic hydrogel was used as a reference. The hydrogels were loaded with 800 µg of dexamethasone and then incubated at 37 °C for 3 min to guarantee the total gelation of materials. Then, 2 mL of PBS at 37 °C was added to the gel layer formed. At the predetermined time, 0.2 mL of the supernatant was extracted and replaced with the same volume of fresh PBS at 37 °C. The amount of dexamethasone released was quantified using UV-Vis spectrophotometry (Thermo Scientific NanoDrop 1000 One Microvolume) at 243 nm.

The release profiles obtained were analyzed according to the mathematical models of Higuchi (Equation (1)), Korsmeyer–Peppas (Equation (2)), Lindner–Lippold (Equation (3)), and Peppas–Sahlin (Equation (4)) according to the equations described below.

The Higuchi model [60,61] describes the release of molecules from materials as a function of the square root of time. It is a time-dependent process based on Fick’s law of diffusion, which is expressed as

(1) $$\frac{M_{t}}{M_{\infty }}=K\cdot t^{1/2}\;$$

where *M_t_/M_∞_* is the amount of drug released on time *t*, and *K* is the release constant.

The Korsmeyer–Peppas model [62,63], also known as a “Power Law” model, describes the drug release from a polymeric system when the release mechanism is not known or when more than one type of phenomenon of drug release is involved.

(2) $$\frac{M_{t}}{M_{\infty }}=K\cdot t^{n}\;$$

where *M_t_/M_∞_* is the amount of drug released on time *t*, *K* is the constant of incorporation of structural modifications and geometrical characteristics of the system (also considered as the release velocity constant), and *n* is the exponent of release (related to the drug release mechanism) as a function of time *t*.

When the drug release process is characterized by an abrupt increase in the initial drug release (burst effect), Lindner and Lippold [64] proposed the following equation

(3) $$\frac{M_{t}}{M_{\infty }}=K\cdot t^{n}+b\;$$

where all terms keep the meaning, and *b* is the burst effect

The Peppas–Sahlin model [65] combines the release due to Fickian diffusion and substrate relaxation mechanisms, resulting in an abnormal release method.

(4) $$\frac{M_{t}}{M_{\infty }}=K_{1}\cdot t^{m}+K_{2}\cdot t^{2m}\;$$

where *M_t_/M_∞_* is the fraction of drug released from the scaffold at time t. *K_1_* is the kinetic constant for the Fickian contribution of drug release, and *K_2_* is the kinetic constant for the substrate relaxation, while *t* represents the release time, and m is the diffusional exponent.

### 4.5. In Vivo Experiments

#### 4.5.1. Animal Models

Ten 12-week-old male C57BL/6Jico mice purchased from Charles River Laboratories (Chatillon-sur-Chalaronne, France) were used. From the group of animals, two of them were taken as a healthy negative control, and the other 8 were injected with collagenase in the right knee to induce osteoarthritis in them [72,73]. The group of 8 mice was subdivided into two groups of 4 mice each in which the prepared materials were injected; the CS1-PF25 hydrogel was applied to one group and the CS1-PF25-TPP hydrogel was applied to the other. Only PBS was applied to the control group. To all the injected samples, as well as the PBS of the control group, the IR-780 iodide dye (NIR-780, Sigma-Aldrich, Amsterdam, The Netherlands) was added to observe the retention time of the materials in the joint. The NIR concentration in the prepared hydrogel formulations and PBS was 50 μg/mL, and the samples volume injected into the mice knee was 10 μL.

#### 4.5.2. In Vivo Images of the Injected Hydrogels

To know the residence time of the materials in the joint, the NIR images were obtained and processed with the PEARL IMPULSE (Li-Cor, Lincoln, NE, USA). The Pearl Impulse has 2 coupled NIR lasers, 700 nm and 800 nm, with filter technology to achieve a stable source of precise and powerful illumination. During the experiment, we always measured and analyzed the signals for all groups with the 800 nm emission filter. The images were acquired after 1, 24, and 48 h of intra-articular injection of the hydrogels and after 7, 14, 21, 28, and 35 days. At each time, the mice were anesthetized with isoflurane balanced with oxygen.

## Figures and Tables

**Table 1:** Composition and nomenclature of the hydrogels.

Sample	CS (%)	PF (%)	TPP (%)
CS1-PF20	1	20	-
CS1-PF25	1	25	-
CS2-PF20	2	20	-
CS2-PF25	2	25	-
CS1-PF20-TPP	1	20	0.4
CS1-PF25-TPP	1	25	0.4

**Table 2:** Summary of the adjustment parameters to the release models for the different hydrogels.

Samples		PF25	CS1-PF25	CS1-PF25-TPP
Model	Parameters			
Higuchi	*K*	12.0 ± 0.6	8.3 ± 0.8	6.7 ± 0.7
	*R^2^*	0.8262	0.2879	0.2402
Korsmeyer–Peppas	*K*	18.6 ± 0.8	20.0 ± 0.2	19.0 ± 0.6
	*n*	0.34 ± 0.01	0.24 ± 0.01	0.23 ± 0.01
	*R^2^*	0.9773	0.9849	0.9712
Lindner–Lippold	*K*	10 ± 2	12 ± 3	10 ± 4
	*n*	0.49 ± 0.07	0.33 ± 0.05	0.33 ± 0.06
	*b*	10 ± 3	8 ± 3	9 ± 4
	*R^2^*	0.9903	0.9888	0.9760
Peppas–Sahlin	*K_1_*	20.6 ± 0.6	20.1 ± 0.2	19.7 ± 0.4
	*K_2_*	0.008 ± 0.001	0.0016 ± 0.0004	0.0008 ± 0.0002
	*n*	0.25 ± 0.02	0.21 ± 0.01	0.19 ± 0.01
	*R^2^*	0.9932	0.9932	0.9885

**Table 3:** Summary of area under the curve values (AUC—area under the curve).

Sample	PBS	CS1-PF25	CS1-PF25-TPP
AUC Total	27,629	36,687	51,562
AUC between 0–1 day	24,050	28,465	38,120
AUC between 1–14 days	3579	7815	12,289

## Data Availability

The data presented in this study are available on request from the corresponding authors.
